# Effect of Curing Temperature on Volume Changes of Alkali-Activated Slag Pastes

**DOI:** 10.3390/ma18051073

**Published:** 2025-02-27

**Authors:** Maïté Lacante, Brice Delsaute, Stéphanie Staquet

**Affiliations:** BATir Department (LGC), Université libre de Bruxelles, 1050 Brussels, Belgium; maite.lacante@ulb.be (M.L.); brice.delsaute@ulb.be (B.D.)

**Keywords:** curing temperature, alkali-activated slag, volume changes, early age

## Abstract

This study investigates the influence of curing temperature (explored at 10 °C, 20 °C, and 30 °C) on the volume changes of alkali-activated slag (AAS) pastes with the aim of expanding existing knowledge on alkali-activated materials (AAMs). The focus was on autogenous and thermal strains, internal relative humidity (IRH), heat flow and cumulative heat, setting times, and workability. The results indicate that increasing the curing temperature to 30 °C reduces autogenous shrinkage, likely due to changes in the elastic modulus and viscoelastic properties, while promoting swelling, especially for higher molarities. The coefficient of thermal expansion (CTE), related to thermal strains, is higher when the curing temperature is increased, but its development is delayed. The IRH is influenced more by the activating solution’s molarity than by curing temperature, although temperature does affect the initial IRH. The study also revealed that higher curing temperatures accelerate chemical reactions and reduce setting times. The initial workability was significantly affected by the solution-to-binder ratio, while higher temperatures decreased workability, especially at higher molarities. These findings contribute to the understanding of how curing temperature influences the durability of AAS pastes, offering insights into optimized construction practices under varying environmental conditions.

## 1. Introduction

Numerous studies have investigated alkali-activated materials (AAMs) and have reported promising outcomes due to their sustainability and performance advantages [1,2,3,4]. However, most of these studies have been conducted at the standard curing temperature of 20 °C. The curing temperature of cementitious materials plays a crucial role in their early-age development, influencing hydration or activation reactions, heat flow, and the subsequent properties of the material. Research on Portland cement (PC) pastes has indicated that the curing temperature can significantly influence, and possibly mitigate, volume changes [5,6,7]. It is crucial to consider how these variations might affect construction practices, especially in different seasonal contexts or geographical locations. In fact, when using cementitious materials in structures, the material is in restrained conditions, resulting in restrained deformation. Therefore, it is important to investigate the autogenous strain and the coefficient of thermal expansion to evaluate the risk of (micro)-cracks in the structure. In addition, the sensitivity of these parameters to temperature is also important in this evaluation due to the potentially high temperatures reached at the core. This research aims to explore the effects of different curing temperatures on autogenous and thermal strains, as well as on heat flow and internal relative humidity. Previous investigations [8] into these properties at 20 °C will serve as reference points for this study.

It is essential to note that the maturity of cementitious materials is influenced by the paste composition and curing temperature. The final magnitude of some properties might be significantly influenced by the curing temperature. Two pastes with identical compositions will exhibit similar mechanical properties at the same maturity level, regardless of their thermal history. The temperature primarily affects the maturity level of the material. This leads to the concept of equivalent age, defined relative to a reference temperature—20 °C in this research. The equivalent age represents the duration required for a sample to achieve the same level of maturity as if cured at the reference temperature, as expressed in Equation (1) [9,10].

(1) $$M\left(t,H\left(t\right)\right)=\int_{0}^{t}K\left(T\left(\tau \right)\right)d\tau =\int_{0}^{t_{eq}}K\left(T_{ref}\right)d\tau =M\left(t_{eq},t_{ref}\right)=K\left(T_{ref}\right)\cdot t_{eq}$$

where the following notations are used:

- M(t,H(t)): maturity at time t for a temperature history H(T);
- K(T): kinetic constant at temperature T;
- T(t): temperature at time t in Kelvin;
- M(teq,Tref): maturity at time t for the reference temperature T_ref_;

From this, it can be concluded that the equivalent age is defined under isothermal curing conditions by Equation (2).

(2) $$t_{eq}=\frac{K(T_{0})}{K(T_{ref})}\cdot t$$

The Arrhenius law (Equation (3)) enables the determination of the kinetic constant, introducing the concept of apparent activation energy.

(3) $$K\left(T\right)=A\cdot exp\left(-\frac{E_{a}}{RT}\right)$$

where the following notations are used:

- *A*: constant of proportionality [1/s];
- *E_a_*: apparent activation energy [J/mol];
- *R*: universal gas constant [J/mol K];
- *T*: temperature [K].

The apparent activation energy ($E_{a}$) reflects the sensitivity of the reaction kinetics of the tested paste to temperature variations [9]. Therefore, the equivalent age can now be calculated using Equation (4).

(4) $$t_{eq}=\int_{0}^{t}exp(-\frac{E_{a}}{R}\left(\frac{1}{T(\tau )}-\frac{1}{T_{ref}}\right)d\tau$$

The apparent activation energy $E_{a}$ is evaluated using the method of superposition [9,10]. The objective is to determine a constant $E_{a}$ value that optimally superimposes two evolution curves of released heat as a function of equivalent age within the interval of heat values [$Q_{inf}$,$Q_{sup}$]. These curves are characterized by distinct temperature histories [9,10]. The value of $E_{a}$ can be determined using the least squares method (see Equation (5)), which minimizes the difference in equivalent age at a specified quantity of released heat $Q_{i}$ [9,10].

(5) $$\min_{E_{a}}\left(\sum_{Q_{i}\in \left[Q_{inf},Q_{sup}\right]}{\left|t_{eq,1i}-t_{eq,2i}\right|}^{2}\right)$$

In addition to reaction kinetics, temperature also influences the internal relative humidity (IRH), which is critical for understanding the evolution of the volume changes. The Kelvin–Laplace equation (Equation (6)) links the pore pressure of fluid to the IRH [11]. When the binder reacts, the IRH decreases over time as the internal solution is consumed during the reaction. This leads to a negative pore pressure, due to which the solution molecules on the meniscus contract, ultimately causing shrinkage [11].

(6) $$p^{\prime }=\frac{-ln(IRH)\cdot R\cdot T}{v\prime }$$

where the following notations are used:

- *p*′: pore pressure of fluid [Pa];
- *IRH*: internal relative humidity [-];
- *R*: universal gas constant [J/mol K];
- *T*: temperature [K];
- *v*′: molar volume of solution [m^3^/mol].

Tests conducted on alkali-activated slag-fly ash (AASF) samples to assess the influence of increased curing temperature on their properties [12] revealed a significant increase in cumulative heat during the first hours of the reaction when the curing temperature is elevated to 40 °C. Analysis of the heat flow shows that this elevated curing temperature accelerates the reactivity of AASF cements. A 40 °C temperature provides the energy necessary to overcome the barrier effect caused by the reaction products precipitating on the particle surfaces. Furthermore, increased temperature enhances the solubility and the diffusivity of the reactants, facilitating the formation of reaction products. These are thermo-activated reactions. Therefore, the study of the apparent activation energy is necessary to evaluate the material’s sensitivity to temperature [13].

It has been observed that as the curing temperature increases (10 °C, 20 °C, and 30 °C), the workability of PC decreases [14]. Moreover, the initial and final setting times for various concretes and mortars made from CEM I, fly ash, blast-furnace slag, and different aggregates are reduced with increased curing temperatures, ranging from 14 °C to 22 °C and up to 40 °C [15]. Research indicates that when the curing temperature is elevated from 20 °C to 40 °C, autogenous shrinkage in AAMs decreases by 25% to 30% [12]. However, from casting time until day 3, autogenous shrinkage initially increases at 40 °C due to a steeper initial slope. This trend is consistent across various sample compositions, including AASF activated with sodium silicate, sodium sulfate, and sodium carbonate. Similar patterns are also noted in cement pastes [16,17].

Further insights from Yeon et al. [11] indicate that temperature variations on PC affect the curvature of the meniscus at the interface between liquid water and vapor, influenced by pore fluid pressure and capillary stress. These are key mechanisms in material deformation. Changes in internal relative humidity or temperature introduce negative pore pressure in the fluid, resulting in the contraction of water molecules at the meniscus. Notably, increases in temperature also elevate the IRH due to pore water expansion, which subsequently increases the meniscus radius (the waterfront moves outward). This phenomenon aligns with the Kelvin–Laplace equation, whereby increased temperature reduces interfacial tension between air and water, further increasing IRH [18].

The relationship between temperature and the coefficient of thermal expansion (CTE) is frequently linked to the internal relative humidity [19,20,21]. However, to the knowledge of the authors, comprehensive studies on the influence of curing temperature on the evolution of the CTE in AAMs at early ages are inexistent.

In conclusion, both internal (related to composition) and external factors (related to curing conditions) considerably affect the development of volume changes in cementitious materials [21]. Among these factors, the curing temperature is a major factor [6]. Therefore, tests have been conducted at 10 °C and 30 °C for this study, comparing the results to the established reference temperature of 20 °C studied by Lacante et al. [8]. The compositions under investigation consist of blast-furnace slag activated with 0.5, 2, and 8 M sodium hydroxide solutions with solution-to-binder ratios (S/B) of 0.5 and 0.8. This research addresses how varying curing temperatures influence key physical and chemical properties of alkali-activated slag (AAS) pastes, including autogenous strain, coefficient of thermal expansion, internal relative humidity, reaction kinetics, and workability. In summary, the aim and the novelty of this paper are to provide a comprehensive understanding of these effects and how curing temperature shapes the behavior of AAS pastes.

## 2. Materials and Methods

### 2.1. Materials and Composition

This paper investigates blast-furnace slag (BFS) as a precursor [7] activated with sodium hydroxide (see Table 1 for chemical composition) on the paste scale. Further details regarding the materials are provided in Lacante et al. [8].

The BFS and NaOH solutions (0.5 M, 2 M, or 8 M) were stored at 10 °C, 20 °C, and 30 °C, corresponding to the curing temperatures used in the experiments. The raw materials were cured in sealed containers for a minimum of 24 h prior to testing [23]. Additionally, precautions were taken to ensure that all devices were set and calibrated to the appropriate curing temperature for at least 24 h before testing. Materials were mixed in accordance with European standard EN 196-1:2016 [24]. Prior to filling the molds, the temperature of the paste is measured to ensure proper temperature control and to accurately estimate the maturation of the material, as described by Equation (4).

The goal of this research is to examine how different curing temperatures influence the properties of alkali-activated slag and the impact of varying the curing temperature on the effects of concentration and solution-to-binder (S/B) ratio. Specifically, two additional curing temperatures, 10 °C and 30 °C, were investigated, and the results were compared to those obtained at 20 °C, reported in Lacante et al. [8]. The reasoning behind these temperature limits is based on the standard EN 1367:2010 [25] and its Belgian supplement NBN B 15-400:2024 [26], which specify that the ambient air temperature must be at least 5 °C during the first 72 h after casting [27]. Furthermore, when the temperature exceeds 25 °C, special measures must be implemented. Consequently, the temperature extremes of 10 °C and 30 °C were selected for investigation, as they represent ±10 °C deviations from the average ambient air temperature of 20 °C. These temperatures are commonly encountered in Belgium and are considered within a realistic range, avoiding extreme temperature conditions. The investigation of the solution-to-binder (S/B) ratio is motivated by its impact on the volume changes development in PC pastes, where the water-to-cement (W/C) ratio plays a key role [28]. In this study, S/B ratios of 0.5 and 0.8 are chosen, as lower ratios result in poor workability, especially when slag is the sole binder [29], while higher ratios increase the risk of bleeding. Additionally, the concentration of the NaOH activator influences the reaction and the volume changes [30]. Molar concentrations of 0.5, 2, and 8 M are selected for investigation, covering a practical range of concentrations while maintaining a factor of four between each. Concentrations below 0.5 M are insufficient for the reaction, and those above 8 M cause excessive heat generation. These compositions have been shown to have good mechanical properties [31]. In addition, the drying shrinkage, the pore structure and the hydraulic conductivity have been investigated by Sirotti et al. [32,33]. The compositions studied are summarized in Table 2.

### 2.2. Devices

#### 2.2.1. Slump Flow

The workability of the paste is assessed following the ASTM C230 standard [34]. The diameter of the paste disc is measured twice immediately after the mixing, with an error margin of ±5 mm. The second measurement is performed at a 90° angle with respect to the first measurement. The assessment of the workability guarantees the quality control of the blast-furnace slag, helping to prevent degradation and ensuring mixing consistency throughout the testing campaign. Because the slump flow test has been performed at each mixing, the amount of tested samples varies between 3 (S05M2, S08M2), 4 (S05M8), and 5 (S05M05, S08M05, S08M8) at 10 °C, 2 (S05M05, S05M8, S08M05, S08M2) and 3 (S05M2, S08M8) at 20 °C, and finally, 3 (S05M05, S05M8, S08M2) and 4 (S05M2, S8M05, S08M2) at 30 °C.

#### 2.2.2. TAM Air Isothermal Calorimeter

The (exothermic) reaction of the materials was studied using the TAM Air isothermal calorimeter (from TA Instruments, New Castle, DE, USA), which satisfies the European norm EN 196-11:2018 [35]. This calorimeter comprises eight channels which can be used simultaneously, although at the same temperature. Each of these channels has two spots. The first one serves to monitor the ampoule filled with about 7.5 g of the material that must be tested. The second spot contains an inert reference ampoule filled with sand, to which the tested ampoule’s heat flow is compared. Each spot has its own heat flow sensor. This allows for erasing noise and increases the stability of the measurement [36].

The heat flow is measured with the device and exhibits two peaks. The first peak characterizes the early dissolution of the slag and is often missed because of its very early occurrence and the ex situ mixing. On the other hand, the second peak is monitored and corresponds to the formation of the reaction products. The period between both peaks is called the induction period [37]. The cumulative heat is computed according to Equation (7), where Q(t) represents the cumulative heat [J/g_slag_] at time *t* [s] and q(τ) [W/g_slag_] denotes the heat flow at time τ [s], with $t_{0}$ being the time zero at which the computation starts. This starting time $t_{0}$ is equal to 0.5 h when the curing temperature is 30 °C while $t_{0}$ is equal to 0.8 h when the curing temperature is 20 and 10 °C. This corresponds to the end of the induction period and the start of the second peak. Therefore, the results of the heat flow are only shown from $t_{0}$ onwards.

(7) $$Q\left(t\right)=\int_{t_{0}}^{t}q\left(\tau \right)\;d\tau \;$$

The mixing is performed ex situ following the European standard EN 196-1:2016 [24]. The filling and the sealing of the ampoules, as well as their placement, is accomplished within the first 10 min after the mix starts. Two ampoules have been tested per composition. The duration of the test depends on the curing temperature. Samples at 10 °C, 20 °C, and 30 °C were monitored for 21, 14, and 7 days, respectively. Regular isothermal calorimetry guarantees quality control of the BFS to prevent degradation and maintains mixing repeatability during the testing campaign.

#### 2.2.3. Vicat Device

The setting times were evaluated with the Vicat device (from Controls, Cernusco, Italy) according to the European standard EN 196-3:2016 [24]. The samples were cured in water during testing and the entire setup was placed into a climatic chamber to maintain curing temperatures of (10 ± 1) °C and (20 ± 1) °C, while an oven was used to achieve (30 ± 1) °C. During each test, a thermocouple was embedded in a dummy sample to monitor the internal temperature, ensuring optimal curing conditions, in addition to considering thermal effects (ageing and maturity) on the setting times. Each composition was tested once, as prescribed by the corresponding standard. Nevertheless, multiple samples from the same mixing were cast to ensure at least two samples for both the initial set and the final set. This allows for taking enough measurements and double-checking the setting time on the other sample.

#### 2.2.4. IRH Probes

The internal relative humidity was monitored with HC2-AW probes (from Rotronic, New York, USA). Similar to the isothermal calorimetry, two specimens were tested and the testing duration was influenced by both the curing temperature and the specific composition being tested. Due to the high IRH of some samples, testing was either initiated later or concluded earlier to prevent damage to the testing device, particularly for compositions with a 0.5 M activator. At 10 °C, only the compositions S05M05, S05M2, and S05M8 were tested to mitigate the risk of damaging the device. Prior to each test, the sensors were calibrated using salt solutions.

#### 2.2.5. Autoshrink

The methodology is based on the AutoShrink [38] method outlined in ASTM standard C1698–09 [39], which has been adapted to study thermal strains in parallel, and thus the coefficient of thermal expansion. For each test, controlled temperature cycles of ±3 °C are applied around the curing temperature, whether it is 10, 20, or 30 °C. The measured strain at time *t* ($\epsilon_{tot}\left(t\right)$ [µm/m]) is the sum of the autogenous strain ($\epsilon_{auto}\left(t\right)$ [µm/m]) and of the thermal strain ($\epsilon_{thermal}\left(t\right)$ [µm/m]), where the latter is characterized by the CTE (α(t) [µm/m/°C]) and the temperature gradient (ΔT(t) [°C]), as shown by Equation (8).

(8) $$\epsilon_{tot}\left(t\right)=\epsilon_{auto}\left(t\right)+\epsilon_{thermal}\left(t\right)=\epsilon_{auto}\left(t\right)+\alpha \left(t\right)\cdot \Delta T\left(t\right)$$

At very early age, it is necessary to decouple the strains, whereas after a certain period of time, the autogenous strain can be considered constant over a small time range, which corresponds to the interval in which the CTE at that time is computed. Therefore, CTE and the autogenous strains are calculated approximately every 2 h. For further details regarding the device and the decoupling of the strains, refer to Delsaute and Staquet [40] and Lacante et al. [8]. At least 2 samples were tested per composition, along with 1 dummy sample for temperature monitoring. As with previous tests, the testing durations varied based on the curing temperature due to the development of maturity. At 10 °C, the samples were monitored for at least 3 weeks, while at 20 °C and 30 °C, the monitoring periods were at least 2 weeks and 1 week, respectively.

## 3. Results and Discussions

### 3.1. Workability

**Figure 1:**
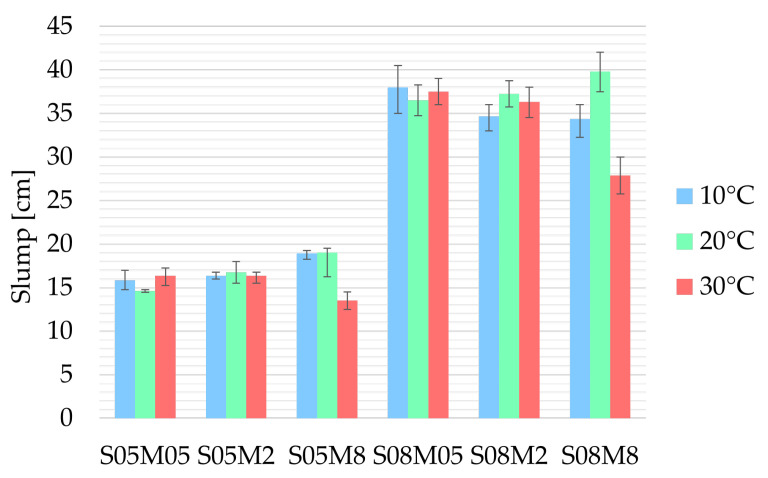
Workability of AAS pastes as a function of different curing temperatures (the error bars represent the minimum and maximum results).

Regardless of the curing temperature, the solution-to-binder (S/B) ratio serves as the primary driving force dictating the spread of the alkali-activated slag (AAS) paste because of the addition of liquid to the material [41] (see Figure 1).

At 30 °C, the paste appears to be less workable for the 8 M concentrations than it is at a curing temperature of 20 °C, a phenomenon also observed in Portland cement (PC) studies [14]. Higher curing temperatures typically lead to the aggregation of cement, the merging of flocs, and the stiffening of the network [42,43], affecting the rheological properties of similar materials [44]. This process accelerates the reaction. In addition, Vázquez et al. [45] showed a decrease in the viscosity of the sodium hydroxide when the temperature is increased. In contrast, the S05M05 and S08M05 compositions seem more workable. However, considering the range of the results per composition (see Figure 1), it may be concluded that temperature does not significantly influence workability during the initial minutes after casting for the 0.5 M compositions. In fact, an average slump diameter across all three curing temperatures of 15.6 and 37.3 cm is obtained for S05M05 and S05M08, respectively. The maximum difference between the average slump and the slump obtained for each of the temperatures is at most 1 cm, which corresponds to the ±5 mm measuring error margin. During the first 10 min of age, the effects of temperature on the reaction are not pronounced [46], aligning with the timeframe in which the test is conducted. Furthermore, increasing the curing temperature enhances the effect of concentration at high S/B ratios, especially for S08M8.

Moreover, reducing the curing temperature to 10 °C does not seem to affect the workability in a clearly identifiable trend.

Overall, the effect of curing temperature on workability appears to be influenced by the concentration of the solution used in the mix. Specifically, at 8 M, workability is significantly reduced when the curing temperature is elevated to 30 °C.

### 3.2. Isothermal Calorimetry

**Figure 2:**
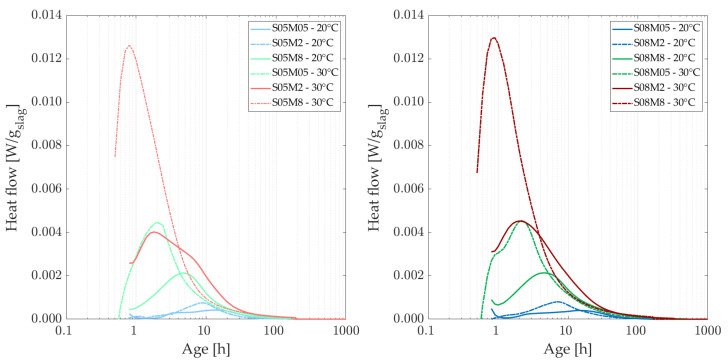
Heat flow of AAS compositions with S/B = 0.5 (**left**) and S/B = 0.8 **(right)**, comparing curing temperatures of 20 and 30 C.

Figure 2 shows the heat flow of the different compositions at the curing temperatures of 20 and 30 °C, normalized by the mass of slag. At a higher curing temperature, it was anticipated that the heat flow peak associated with the formation of reaction products would occur sooner than at 20 °C [47]. Observations reveal that the peak occurs approximately 2 h earlier and is significantly higher at 30 °C, with peak values nearly double those observed at 20 °C. At a curing temperature of 30 °C, the peak heat flow values are observed to be 1.8 to 2.9 times higher than those recorded at 20 °C, see Table 3 (where the peak variations (increase) from one curing temperature to another is shown per composition). Additionally, for the 8 M concentration, the heat flow peaks occur before the end of the first hour at 30 °C. Furthermore, the impact of increasing the S/B ratio becomes more pronounced at elevated temperatures. Higher temperatures accelerate the cumulative heat generation and enhance the reactivity of AAMs, providing sufficient energy to overcome the barrier effect caused by reaction products precipitating on particle surfaces. Additionally, the formation of reaction products is facilitated by increased solubility and diffusivity at higher temperatures [44,48,49].

**Figure 3:**
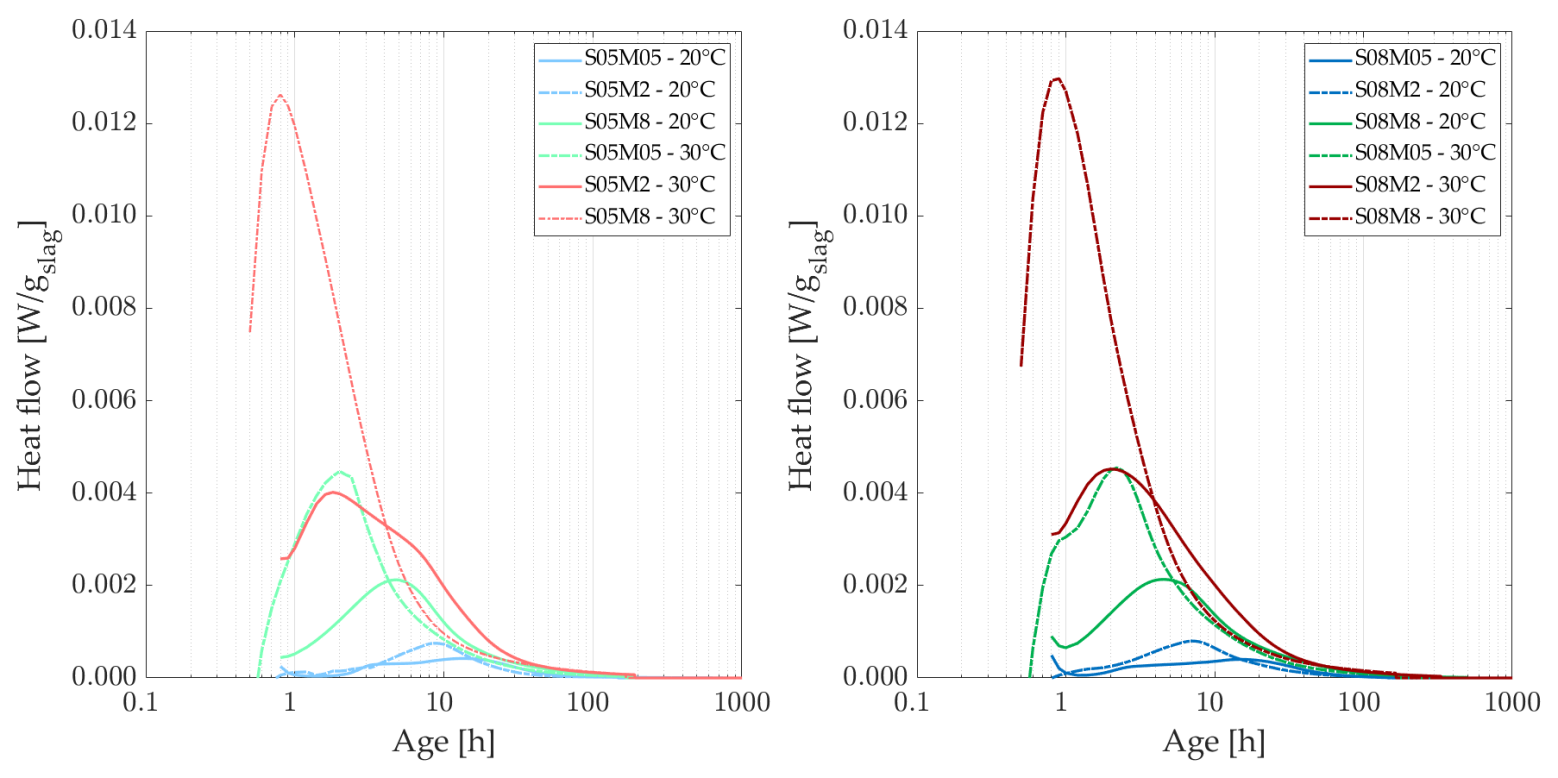
Heat flow of AAS compositions with S/B = 0.5 (**left**) and S/B = 0.8 (**right**), comparing curing temperatures of 10 and 20 C.

Figure 3 shows the heat flow of the different compositions at the curing temperatures of 10 and 20 °C, normalized by the mass of slag. At a curing temperature of 10 °C, the second peak occurs later, indicating a longer induction period. Additionally, lower concentrations of the activator result in later observations of this second peak. Interestingly, the 8 M compositions demonstrate the most significant amplitude variations across different curing temperatures, whereas the 2 M and 0.5 M compositions exhibit relatively similar trends in heat flow. Overall, as the curing temperature decreases, the heat flow curves start to rise after 3 h for the most reactive compositions, compared to an increase observed before 1 h at 20 °C. This indicates that a decrease in curing temperature slows down the reaction in general.

Table 4 shows the peak variation (increase) due to an increase in the activator, for each temperature and each S/B. The factors between the summits of the peaks of the heat flow evolve inversely with molarity at each curing temperature. Specifically, the factor between lower molarities is higher than that between higher molarities.

**Figure 4:**
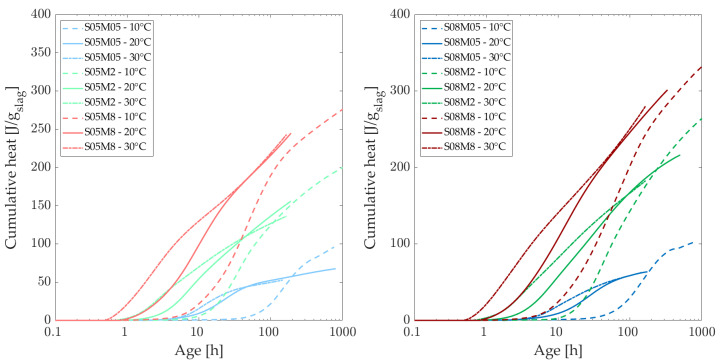
Cumulative heat of AAS compositions at different curing temperatures with S/B = 0.5 (**left**) and S/B = 0.8 (**right**).

Figure 4 shows the results of the cumulative heat of the compositions at different curing temperatures, normalized by the mass of slag. The cumulative heat is the integration of the heat flow over time. The S/B ratio has minimal influence on the heat flow. However, its impact is more noticeable on the cumulative heat: a higher S/B ratio results in greater cumulative heat, because increased activator availability enhances the heat generation during the reaction [50]. The effect of S/B is more pronounced at 30 °C compared to 20 °C and 10 °C, with the separation between curves for different S/B ratios occurring more rapidly as the curing temperature rises.

For the 0.5 M compositions, although the second peak of the heat flow at 10 °C is smaller in magnitude than at 20 °C, it lasts longer. At 20 °C, this peak occurs between 1 h and 100 h, while at 10 °C, it spans from 20 h to 400 h, resulting in a higher cumulative heat. Notably, this significant difference is not observed between 20 °C and 30 °C. Similarly to the 0.5 M results, more heat is released during the first 10 h at 30 °C compared to 20 °C and 10 °C for the 2 M compositions. At 30 °C, the cumulative heat curves remain superimposed during the first three hours, while at 20 °C, this similarity persists for about 10 h, and at 10 °C, it lasts for 40 h. For the 8 M compositions at curing temperatures of 20 °C and 30 °C, the heat flow curves remain superimposed for a similar duration, contrasting with the behaviors observed at lower molarities. However, at 10 °C, the curves remain superimposed for an extended period of time of about 80 h. This superposition is influenced by both the molarity and the curing temperature. Generally, compositions with an 8 M activator exhibit a faster reaction development, making it challenging to evaluate the difference in curing temperature at very early ages.

Temperature and molarity exert the most significant influence on the heat flow of AAS pastes, both contributing to an increase in heat flow. However, this influence seems to be the opposite for cumulative heat. The S/B ratio has a minor impact on the heat flow, primarily observable in the cumulative heat curves, where the curves diverge over time due to the S/B ratio. Additionally, the curing temperature enhances the effect of molarity. It is a thermo-activated reaction. As temperature and molarity increase, the rate of cumulative heat evolution accelerates [12].

Since cumulative heat is the integration of the area under the heat flow curve over time, we can expect the evolution at 30 °C to occur faster without clearly surpassing the results at 20 °C. This is because the peak at higher temperatures is narrower than that at 20 °C, especially when considering that it is represented on a logarithmic scale.

#### 3.2.1. Ultimate Heat

Table 5 presents the ultimate heat $Q_{\infty }$ for all compositions as a function of different curing temperatures. The ultimate heat represents the total amount of heat a reaction can produce until completion. A lower curing temperature corresponds to a higher ultimate heat. However, results at 20 and 30 °C are very similar. S05M8 and S08M8 also exhibit relatively similar $Q_{\infty }$ across different curing temperatures, despite missing part of the second heat flow peak and starting the computation of the cumulative heat earlier at 30 °C.

The $Q_{\infty }$ difference between 10 °C and 20 °C ranges from 1.06 to 2.09, while the difference between 20 °C and 30 °C is only 0.95 to 1.27 (see Table 5).

(9) $$DOR\left(t\right)=\frac{Q(t)}{Q_{\infty }},$$

Using the ultimate heat, the degree of reaction (DOR) can be computed for each composition by using Equation (9), where Q(t) denotes the cumulative heat at time *t* and $Q_{\infty }$ represents the corresponding ultimate heat. This approach facilitates the interpretation of the data in terms of reaction progress.

#### 3.2.2. Apparent Activation Energy

The determination of apparent activation energy E_a_ is necessary for the calculation of the equivalent age relative to 20 °C. This relates to the maturity of the materials. The apparent activation energy is not necessarily constant over time. However, a mean value can be calculated during the early-age period [10]. The activation energy for AAS has already been computed in [8] and is reported in Table 6. Notably, the maturity of AAS is more sensitive to temperature changes than that of PC materials [51].

### 3.3. Setting Time

**Figure 5:**
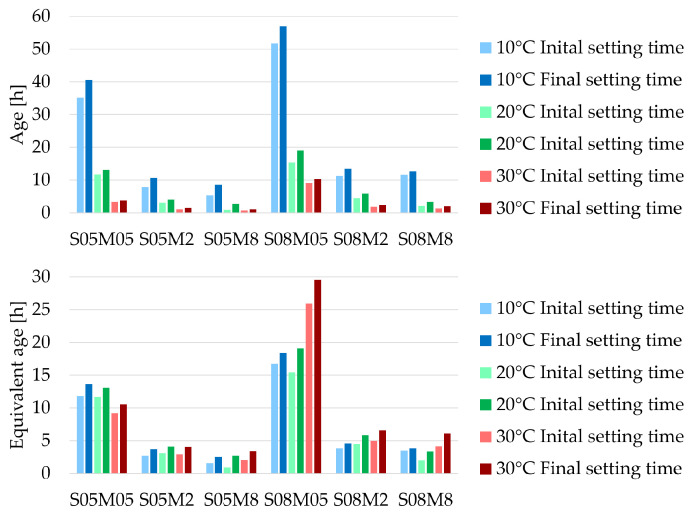
Setting times of AAS pastes at different curing temperatures, determined with the Vicat criterion.

As expected, the setting times decrease with increasing curing temperature [15] (see Figure 5). Table A1 in the Appendix A summarizes all the setting times of Figure 5, determined with the Vicat criterion. The isothermal calorimetry results revealed a significantly higher reaction and rate of reaction. This combination of accelerated kinetics and increased heat release at very early age contributes to the overall quicker setting of the material.

However, in terms of maturity, which relates to equivalent age and the apparent activation energy, the setting times generally appear to increase over time when the curing temperature increases. A relatively good correlation can be observed between the results at 10 °C and 20 °C with a maximum difference in (equivalent) age of 73% (S05M8) for the initial set (with an average of 30%) and a maximum difference of 22% (S08M2) for the final set (with an average difference of 10%). However, the results at 30 °C show significant deviation, with a maximum difference in (equivalent) age of 129% with respect to the results at 20 °C (S05M8) for the initial set (with an average of 56%) and a maximum difference of 82% (S08M8) for the final set (with an average difference of 32%). This discrepancy may be attributed to “time conversion” as well as to the increased apparent activation energy. For example, the setting times for the 2 M and 8 M samples are below 6 h at 20 °C and below 2 h and 30 min at 30 °C. Given that mixing and conducting the slump test require time, followed by the pouring of the Vicat tests, the entire process of sample placement also takes additional time. As a result, at 30 °C, the paste is mixed, adjusted, and modified for a longer proportion of the setting times compared to 20 °C or 10 °C. For instance, if the entire sample placement process takes 20 min, the extended time spent handling the mix at 30 °C (up to 47% of the setting time) represents a much larger proportion of the total setting time compared to 10 °C (up to 6% of the setting time). This increased manipulation may prevent the mix from properly initiating its setting process, explaining why the equivalent age setting times at 30 °C are higher, while those at 20 °C and 10 °C are more similar.

### 3.4. Internal Relative Humidity

**Figure 6:**
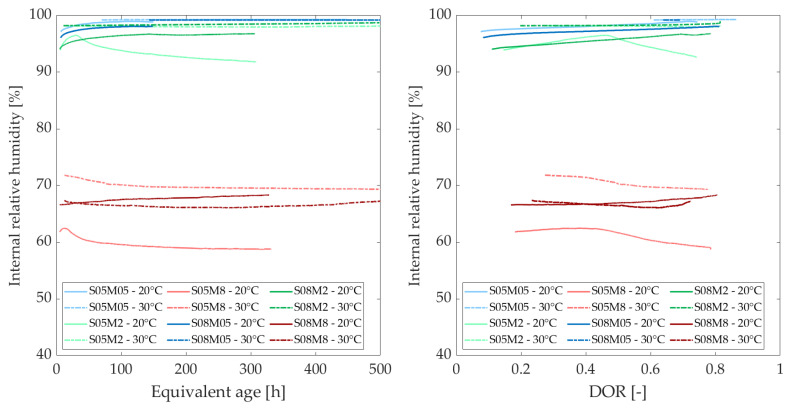
Internal relative humidity as a function of the equivalent age (**left**) and degree of reaction (**right**), comparing curing temperatures of 20 and 30 C.

**Figure 7:**
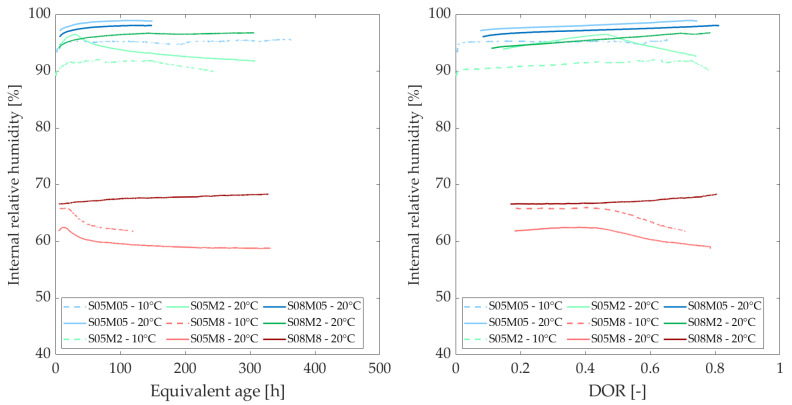
Internal relative humidity as a function of the equivalent age (**left**) and degree of reaction (**right**), comparing curing temperatures of 10 and 20 C.

Figure 6 and Figure 7 show the evolution of the internal relative humidity (IRH) of the AAS pastes at different curing temperatures, as a function of the equivalent age (left) and as a function of the degree of reaction (right).

All graphs display three groups of curves corresponding to the activating solutions used in the compositions. The concentration of activating solution is the most significant factor influencing IRH, surpassing the effects of S/B and curing temperature. In addition, a higher drop is expected for compositions with lower S/B [52]. Initial observations suggest that higher molarity results in lower IRH values. Notably, the values obtained for the 8 M compositions do not fall within the same range as those for cement-based materials [6,18]. To better understand this phenomenon, the IRH of the solutions was measured. Additionally, since temperature appears to influence the range of IRH values, these effects will be analyzed.

**Figure 8:**
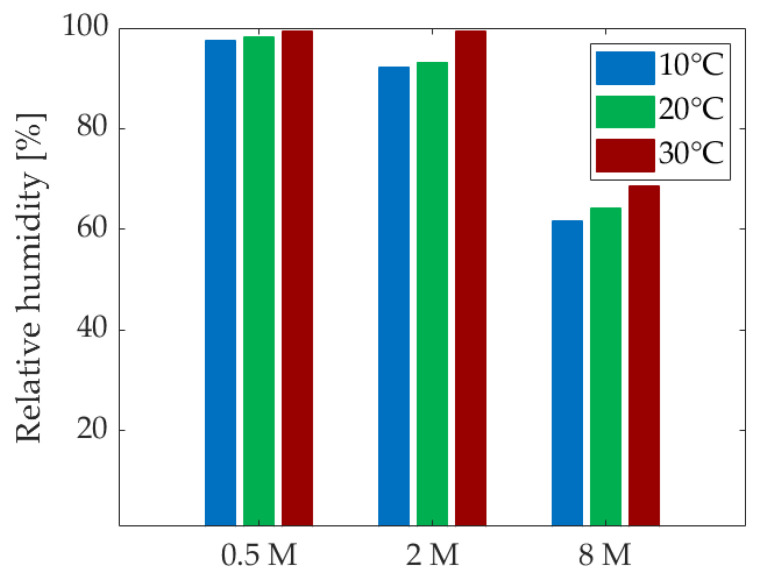
Relative humidity of the activating solution at different curing temperatures.

The relative humidity (RH) of the different solutions is illustrated in Figure 8. Notably, at low molarities, the solution exhibits approximately the same relative humidity as water. As the molarity of the solution increases, the IRH decreases [53]. This follows a similar mechanism to that of calibration solutions for the device, where salts are dissolved in distilled water until saturation, producing a known relative humidity. In this case, dissolving solid NaOH in distilled water results in a specific relative humidity. The initial IRH of a paste immediately after mixing is equal to the RH of the solution. This is why concrete typically approaches 100% at early ages before gradually decreasing, yet remaining above 75% [54]. The low IRH of the 8 M NaOH explains why the IRH of the 8 M compositions is already quite low after just 24 h. As expected from Madge [53], the relative humidity of each solution appears to increase with the increase of the temperature.

**Figure 9:**
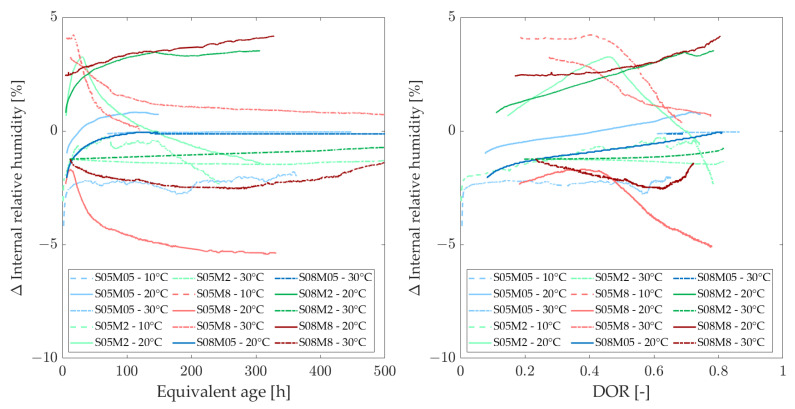
Internal relative humidity with respect to RH of the activator as a function of the equivalent age (**left**) and degree of reaction (**right**) at different curing temperatures.

The initial IRH of a paste, immediately after mixing, is equal to the relative humidity of the activating solution. Therefore, Figure 9 illustrates the variation of the IRH of the pastes with respect to the relative humidity of the activating solution, as described by Equation (10).

(10) $$\Delta IRH_{Composition}\left(t\right)=IRH_{Composition}\left(t\right)-RH_{ActivatingSolution}$$

The increase in IRH over time is attributed to the ongoing reaction and the decreasing concentration of ions in the pore solution [55,56]. During the reaction, ions in the solution combine with reaction products, leading to a decrease in the total ion concentration. As ions are consumed, the total amount of ions in the pore solution decreases and the relative humidity of the pore solution rises, which in turn increases the overall IRH of the sample. Conversely, the decrease in IRH is closely linked to self-desiccation, as all of the solution is consumed and drawn into the paste due to pore pressure [56]. Typically, at 20 °C, both phenomena occur when the S/B ratio is 0.5, whereas only the increase in IRH is observed at an S/B ratio of 0.8.

Overall, the trend in the development of IRH for each composition does not appear to be significantly affected by the curing temperature, particularly in terms of degree of reaction. Generally, an increase in curing temperature seems to lead to a gradual increase in IRH or a smaller decrease in IRH compared to lower curing temperatures. However, for the S08M8 composition, an initial decrease is observed, followed by an increase. Variations in temperature significantly affect the initial IRH values, which correlate with the relative humidity of the alkali activators. Specifically, an increase in temperature raises the RH of the solution [53], consequently increasing the IRH of the material. Additionally, as water expands with increasing temperature, the surface tension decreases, resulting in a larger radius of meniscus curvature [20].

### 3.5. Autogenous Strain

**Figure 10:**
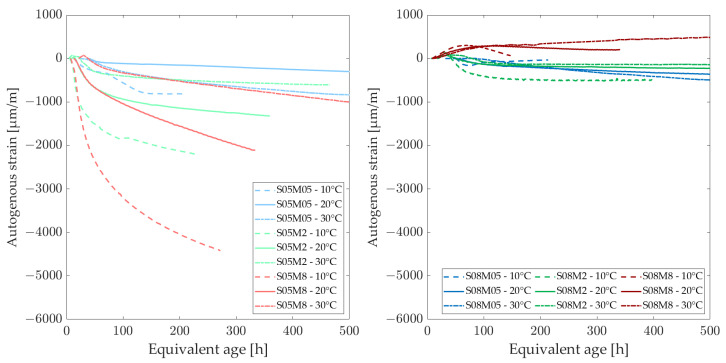
Autogenous strain at different curing temperatures as a function of the equivalent age.

**Figure 11:**
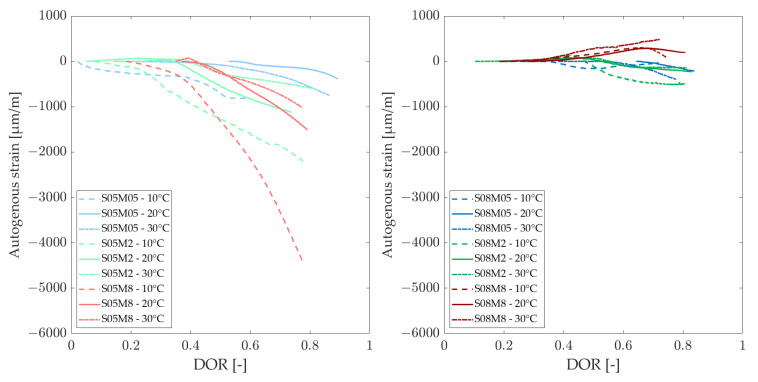
Autogenous strain at different curing temperatures as a function of the degree of reaction.

**Figure 12:**
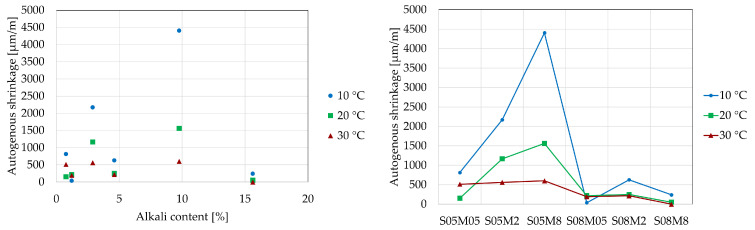
Amount of autogenous shrinkage [µm/m/°C] as a function of alkali content (**left**) and composition (**right**).

**Figure 13:**
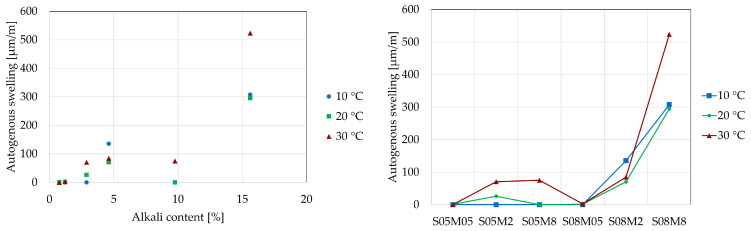
Amount of autogenous swelling [µm/m/°C] as a function of alkali content (**left**) and composition (**right**).

The development of autogenous strain can generally be divided into three stages: chemical shrinkage, swelling, and self-desiccation shrinkage. In this study, the strains are set to zero at the final set, determined using the knee-point method for each composition at each curing temperature [57] because the Vicat final setting time might not be the optimal choice for these materials, as shown by Lacante et al. [8] and by Naqi et al. [58]. Figure 10 and Figure 11 show the autogenous strain results for the different AAS compositions studied in this paper at different curing temperatures, as a function of the equivalent age and degree of reaction, respectively. Table 7 and Table 8 relate the amount of swelling and shrinkage that each composition exhibits at each curing temperature based on Figure 10 and Figure 11. Figure 12 and Figure 13 show the amount of swelling as a function of the composition and the alkali content.

Similar to the observations at 20 °C, the 0.5 M and 2 M compositions exhibit the same trend regarding the S/B ratio at 30 °C. However, the 8 M compositions display different behavior. Notably, the S05M8 composition shows some swelling, which was not observed at 20 °C. Additionally, the S08M8 composition demonstrates no signs of shrinkage, exhibiting only swelling. At early ages, the S08M8 composition at 20 °C exhibited minimal shrinkage, but this was not entirely absent, unlike at the higher curing temperature. In comparison with 20 °C, the autogenous shrinkage is reduced at 30 °C [59,60], while the swelling is enhanced and prolonged in terms of equivalent age and of degree of reaction. The total amount of swelling is increased, while the autogenous shrinkage is reduced for most compositions when increasing the curing temperature to 30 °C. In general, Uppalapati [12] found a decrease in the autogenous shrinkage of AAMs by 25% to 30% when the temperature was elevated from 20 °C to 40 °C. In the case of the present research (excluding S08M8, which did not present any shrinkage at 30 °C, and S05M05, whose shrinkage increased by more than double), a decrease of (56.7 ± 4.8)% for S/B = 0.5 and a decrease of (12.3 ± 0.9)% for S/B = 0.8 was observed when increasing the temperature from 20 °C to 30 °C.

At 10 °C, only the S08M2 and S08M8 compositions exhibit swelling while high shrinkage is observed for all compositions. The S08M8 composition also demonstrates slight swelling between 10 and 60 h. In contrast, the S08M05 composition exhibits shrinkage at very early ages, followed by a swelling stage. For the remaining compositions, no swelling is observed. For the S05M2, swelling is almost noticeable, but it does not overcome shrinkage. The total amount of swelling seems to be higher for the compositions that exhibit swelling (S08M2 and S08M8) and non-existent for the other compositions, while autogenous shrinkage is amplified for most compositions (except for S08M05) when decreasing the curing temperature to 10 °C.

On the one hand, autogenous swelling has been linked to the following causes:

- Reaction products: hydrotalcite group minerals, which are crystalline reaction products, are formed during the reaction and might cause swelling, similar to how ettringite is responsible for swelling in PC pastes. However, they are less expansive and less abundant [61].
- IRH correlation: IRH and autogenous strain are intimately linked. An increase in the IRH results in an increase in autogenous strain, similar to autogenous shrinkage [62]. When the IRH increases, it results in a larger Kelvin radius of the pore, which reduces the pressure and thus induces swelling.
- Solution reabsorption: the solution is reabsorbed during this phase, leading to an increase in volume [63].

On the other hand, the autogenous shrinkage has been explained by the following causes:

- Capillary pressure: dense pore structure compared to PC pase. This induces a larger surface tension in the pore solution. The saturation degree is higher and results in a higher magnitude of capillary pressure [21].
- Deformability: the C-A-S-H gels present in AAS paste are highly viscoelastic, which increases deformability [61].
- Polymerization: the distances between the solid particles are reduced because of the poly-condensation between adjacent gel units during the solid network formation [12].
- Disbalance of forces: as the attractive force between gel particles does not change, a reduction in the concentration of ions present in the pore solution results in a decrease in the repulsive steric-hydration force [64].

A higher IRH reduces autogenous shrinkage by mitigating self-desiccation. An increase in curing temperature results in a lower Kelvin radius at early age, as the elevated temperature accelerates the reaction rate and the self-desiccation process. Higher temperatures lead to a higher coarse porosity in the material [65,66]. Typically, low Kelvin radii of the pores would suggest high capillary tension in the pore fluid, which could lead to significant autogenous shrinkage. However, this decrease in shrinkage can be explained by the material’s elastic modulus; studies have shown that the elastic modulus at higher curing temperatures is higher. This increase in the elastic modulus may account for the lower autogenous shrinkage observed at elevated temperatures, despite the presence of higher capillary tension in the pore fluid. It is hypothesized that additional factors, such as creep, relaxation, and micro-cracking, also contribute to the reduction in autogenous shrinkage at higher curing temperatures [12]. The adhesion between adjacent C-A-S-H gels is improved and the development of the strength is accelerated when higher temperatures are used for curing. This brings improvements to the C-A-S-H gels’s stability [67]. In addition, the increase in curing temperature might lead to a decrease of the C-A-S-H gels’s visco-elastic compliance, thus leading to an increase in the deformability of the material [59,68]. Moreover, with regard to the deformability of the material, an elevated curing temperature results in more uniform C-A-S-H gels with a reduced cross-linking degree, which tends to enhance shrinkage and reduce swelling [1].

### 3.6. Coefficient of Thermal Expansion

**Figure 14:**
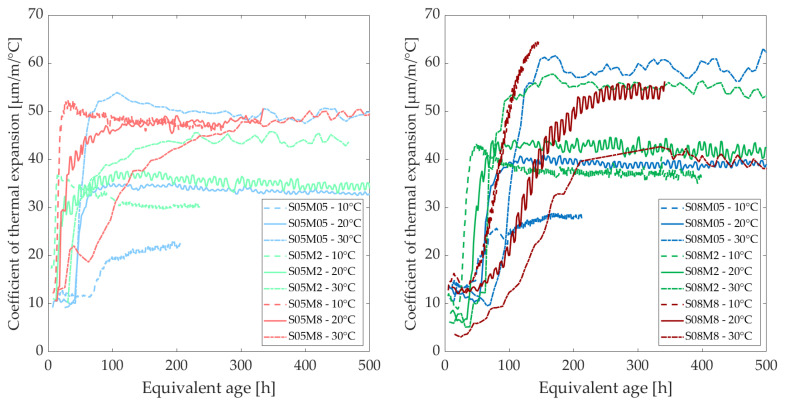
Coefficient of thermal expansion at different curing temperatures as a function of the equivalent age.

**Figure 15:**
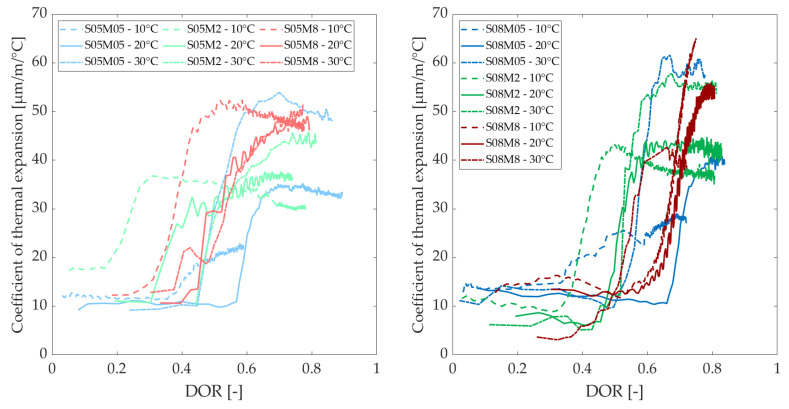
Coefficient of thermal expansion at different curing temperatures as a function of the degree of reaction.

Figure 14 and Figure 15 show the evolution of the coefficient of thermal expansion (CTE) of the compositions at different curing temperatures, as a function of the equivalent age and degree of reaction, respectively. Table 9 shows the CTE values for each composition at 200 h. The curing temperature has a significant effect on the CTE of alkali-activated slag pastes, leading to an increase in CTE for the 2 M and 0.5 M compositions while delaying its development for all compositions. The curing temperature is inversely proportional to the rate of development. On average, the CTE at 30 °C is higher than the one at 20 °C. After 200 h at 20 °C, the CTE ranges between 34 and 50 µm/m/°C, whereas at 30 °C, it ranges between 42 and 58 µm/m/°C (see Table 9). The CTE of the 0.5 M and 2 M compositions are about 1.5 and 1.2 times higher than their CTEs at 20 °C. An increase by 1.1 and 1.3 can be expected when the S/B is increased from 0.5 to 0.8 for the 0.5 M and 2 M compositions, respectively, compared to 1.1 and 1.2 at 20 °C. Conversely, the 8 M compositions exhibit relatively similar CTEs, which is also the case at 20 °C. Finally, an increase in the molarity from 0.5 M to 2 M, and from 2 M to 8 M, leads to CTEs that are approximately 1.1 and 1.2 times lower, respectively, while they increase by about 1.1 and 1.2 at 20 °C.

Interestingly, while the CTE tends to increase with higher molarity at 10 °C and 20 °C, this trend reverses at 30 °C. However, a consistently higher CTE is observed with elevated S/B. At 10 °C, the CTE stabilizes between 20 and 40 µm/m/°C for the 0.5 M and 2 M compositions, while it is somewhat higher at 20 °C. In contrast, the 8 M compositions stabilize at much higher values. The S05M8 stabilizes at 50 µm/m/°C at all three temperatures, whereas the S08M8 shows a more substantial increase, likely stabilizing between 65 and 70 µm/m/°C. Additionally, the shape of the S08M8 curve at 20 °C is similar to that at 10 °C. At lower curing temperatures, the stabilization time for the 8 M and 2 M compositions is reduced. The CTE of the 0.5 M and 2 M compositions is approximately 1.5 and 1.2 times lower than their CTEs at 20 °C. When S/B is increased from 0.5 to 0.8, an increase of approximately 1.3, 1.1, and 1.4 times can be expected for the 0.5 M, 2 M, and 8 M compositions, respectively. Additionally, increasing the molarity from 0.5 M to 2 M, and from 2 M to 8 M, results in CTE values that are approximately 1.3 and 1.7 times higher, respectively.

In PC pastes, it has been observed that a decrease in IRH results in an increase in CTE (when IRH > 60%, before which it decreases, exhibiting a bell-shaped curve) [21,69]. For AAMs, this is only the case for the effect of the concentration of the activator: increasing the concentration decreases the IRH and increases the CTE. However, when looking at the curing temperature, its increase results in higher IRH and the CTE also increases, while a lower CTE is observed for PC [6,19,20].

Overall, the CTE of AAMs is higher than that of Portland cement, indicating that AAMs undergo more thermal deformation than PC [70]. In addition, the CTE is also much more sensitive to the curing temperature than the CTE of PC. According to the study conducted by Li et al. [71], the curing temperature (18 °C to 25 °C) does not seem to affect the CTE of PC concrete. This difference with regard to PC compositions must be taken into account in construction. Prefabrication procedures should be adapted if such materials are used. Indeed, higher temperatures are often used during curing in prefabrication. Consequently, when the material cools down, high thermal strains are restrained, resulting in thermal stresses and increasing the risk of (micro-)cracking.

**Figure 16:**
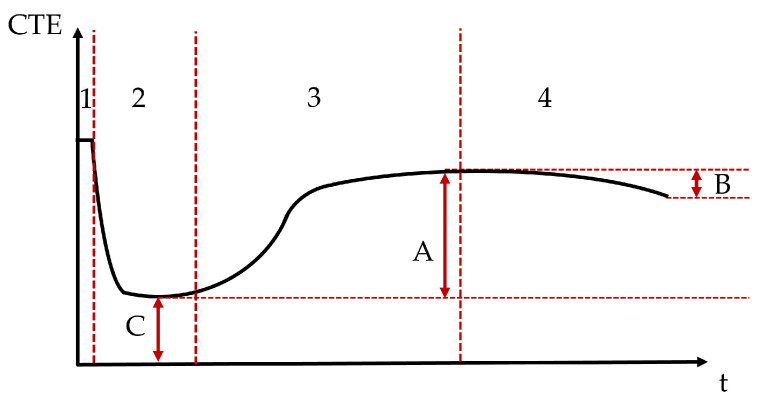
Evolution stages of the CTE.

The evolution of the CTE can be divided into four stages [21] (see Figure 16). During the first stage, the paste remains in a plastic state, exhibiting a relatively high CTE [72], which is largely influenced by the water content. As the paste progresses into the second stage, the CTE decreases sharply as it begins to set, reaching its lowest point (characterized by **C**), which corresponds to the CTE of the solid skeleton. In the third stage, the CTE increases once again by an amount **A** as the internal humidity of the paste decreases [73]. Finally, in the fourth stage, the CTE stabilizes, but a small decrease **B** can be expected [51], possibly due to an increase in the elastic modulus over time [31].

## 4. Conclusions

The effect of curing temperature on volume changes in AAS pastes was investigated in this paper. The following conclusions can be drawn:

- The development of autogenous strain in AAS pastes is influenced by curing temperature, with variations in swelling and shrinkage observed across the different compositions. At higher curing temperatures, autogenous shrinkage is generally reduced, possibly due to an increased elastic modulus and changes in the material’s viscoelastic properties. Swelling, on the other hand, is enhanced at elevated temperatures, particularly for higher molarities, and is linked to factors like IRH, solution reabsorption, and reaction products.
- The CTE is significantly influenced by curing temperature, with higher temperatures generally leading to increased CTE, especially for the 0.5 M and 2 M compositions. Elevated curing temperatures also delay the development of CTE. Parameter C, related to the CTE of the solid skeleton, is on average equal to 10.4 µm/m/°C, similar to that of PC.
- The IRH of these pastes is primarily influenced by the molarity of the activating solution, with higher molarities leading to lower IRH values. The decrease in IRH over time is attributed to self-desiccation, while increases are due to the decrease of the ions during the ongoing chemical reactions. While curing temperature affects the RH of the solution and thus the initial IRH of the paste, its impact on IRH development over time is less pronounced.
- Increasing the curing temperature accelerates the reaction and reduces setting times. However, at 30 °C, extended handling during sample preparation may delay the setting process in terms of equivalent age, leading to longer equivalent setting times compared to 20 °C and 10 °C.
- Elevating the curing temperature leads to a faster reaction with a higher amount of heat production. It is a thermo-activated reaction. Thanks to the cumulative heat, the ultimate heat was deduced: it reaches higher values when the curing temperature is lower.
- The workability of AAS pastes is primarily influenced by the S/B ratio, with curing temperature affecting workability more at higher concentrations. At 30 °C, workability decreases, especially for 8 M NaOH, while lower temperatures also reduce workability.

This new understanding of the effect of curing temperature on volume changes in AAMs, and more interestingly, on the coefficient of thermal expansion and autogenous strain, will improve the accuracy of the material behavior model used for computation by finite elements in massive concrete structures. Understanding the coefficient of thermal expansion and its dependence on curing temperature is crucial for the modeling and improvement of models of such structures, especially at early age.

Future work will focus on the evaluation of the coefficient of thermal expansion as time approaches t = *∞*, as well as its variation with respect to different relative humidities. Other mitigation strategies, such as the use of substitution materials (limestone filler and metakaolin), will be investigated. Attention should also be given to alternative methods to determine setting time at 30 °C because of the long preparation time needed. For example, this parameter can be determined thanks to continuous ultrasonic measurements [74].

## Figures and Tables

**Table 1:** Chemical composition [%] of BFS in mass percent from X-ray fluorescence spectroscopy [22].

SiO_2_	Al_2_O_3_	Fe_2_O_3_	CaO	K_2_O	MgO	TiO_2_	SO_3_	Na_2_O	BaO	MnO
33.30	12.30	0.39	40.80	0.67	7.84	1.29	2.30	0.44	0.31	0.36

**Table 2:** Studied composition and their mixed proportions.

Composition	S/B [-]	NaOH Concentration [M]	Curing Temperature [°C]
S05M05—10 °C	0.5	0.5	10 °C
S05M05—20 °C	0.5	0.5	20 °C
S05M05—30 °C	0.5	0.5	30 °C
S05M2—10 °C	0.5	2	10 °C
S05M2—20 °C	0.5	2	20 °C
S05M2—30 °C	0.5	2	30 °C
S05M8—10 °C	0.5	8	10 °C
S05M8—20 °C	0.5	8	20 °C
S05M8—30 °C	0.5	8	30 °C
S08M05—10 °C	0.8	0.5	10 °C
S08M05—20 °C	0.8	0.5	20 °C
S08M05—30 °C	0.8	0.5	30 °C
S08M2—10 °C	0.8	2	10 °C
S08M2—20 °C	0.8	2	20 °C
S08M2—30 °C	0.8	2	30 °C
S08M8—10 °C	0.8	8	10 °C
S08M8—20 °C	0.8	8	20 °C
S08M8—30 °C	0.8	8	30 °C

**Table 3:** Factors representing the variation (increase) in heat flow peaks due to the increase in curing temperature per composition.

Curing Temperature	10 °C to 20 °C	20 °C to 30 °C	10 °C to 30 °C
S05M05	3.4	1.8	6.1
S05M2	3.0	2.1	6.3
S05M8	3.9	3.1	12.3
S08M05	3.0	2.0	5.9
S08M2	3.0	2.1	6.4
S08M8	4.0	2.9	11.6

**Table 4:** Factors representing the variation (increase) in heat flow peaks due to the increase in the concentration of the activator, per temperature and per solution-to-binder ratio.

Curing Temperature	10 °C	20 °C	30 °C
**Solution-to-Binder**	**0.5 | 0.8**	**0.5 | 0.8**	**0.5 | 0.8**
0.5 M to 2 M	5.8 | 5.2	5.0 | 5.3	6.0 | 5.7
2 M to 8 M	1.4 | 1.6	1.9 | 2.1	2.8 | 2.9

**Table 5:** Ultimate heat $Q_{\infty }$ [J/g_slag_] of AAS as function of the curing temperature.

Composition	10 °C	20 °C	30 °C
S05M05	150.39	75.07	60.97
S05M2	243.78	210.67	166.89
S05M8	325.63	317.13	310.07
S08M05	128.39	78.53	80.42
S08M2	333.21	263.05	223.95
S08M8	400.43	376.97	370.76

**Table 6:** Apparent activation energy *E_a_* of each composition [8].

Composition	S05M05	S05M2	S05M8	S08M05	S08M2	S08M8	Average
*E_a_* [kJ/mol]	74.18	71.99	83.63	69.08	73.08	81.05	75.50

**Table 7:** Amount of autogenous swelling [µm/m/°C] for each composition in function of curing temperature.

Composition	10 °C	20 °C	30 °C
S05M05	0.00	0.99	0.00
S05M2	0.00	26.16	70.77
S05M8	0.00	0.00	75.26
S08M05	0.00	1.47	2.74
S08M2	135.23	70.58	84.79
S08M8	307.64	295.04	692.35

**Table 8:** Amount of autogenous shrinkage [µm/m/°C] for each composition as function of curing temperature.

Composition	10 °C	20 °C	30 °C
S05M05	812.19	152.45	512.11
S05M2	2169.91	1166.19	561.80
S05M8	5165.90	1560.68	600.75
S08M05	38.77	217.18	192.48
S08M2	626.57	248.81	216.01
S08M8	239.80	49.88	0.00

**Table 9:** Coefficients of thermal expansion [µm/m/°C] at 200 h for each composition as a function of the curing temperatures.

Composition	10 °C	20 °C	30 °C
S05M05	21.53	34.30	50.09
S05M2	30.06	36.39	43.59
S05M8	45.99	49.09	42.32
S08M05	28.42	38.92	57.59
S08M2	34.21	43.14	55.21
S08M8	64.89	48.87	42.38

## Data Availability

Data are contained within the article.

## Abbreviations

The following abbreviations are used in this manuscript:

AAMs	Alkali-activated materials
AAS	Alkali-activated slag
CTE	Coefficient of thermal expansion
DOR	Degree of reaction
IRH	Internal relative humidity
M	Mol/L
PC	Portland cement
q	Heat flow
Q	Cumulative heat
RH	Relative humidity
S/B	Solution-to-binder mass ratio

## Appendix A

Table A1 summarizes the setting times displayed in Figure 5.

**Table A1:** Setting times [h] of AAS pastes.

Composition	S05M05	S05M2	S05M8	S08M05	S08M2	S08M8
**Setting Time**	**Initial | Final**	**Initial | Final**	**Initial | Final**	**Initial | Final**	**Initial | Final**	**Initial | Final**
10 °C (t)	35.17 | 40.62	7.87 | 10.72	5.38 | 8.58	51.78 | 56.97	11.28 | 13.48	11.63 | 12.70
10 °C (t_eq_)	11.81 | 13.64	2.71 | 3.69	1.56 | 2.49	16.72 | 18.39	3.83 | 4.57	3.51 | 3.83
20 °C (t)	11.68 | 13.08	3.08 | 4.05	0.90 | 2.72	15.40 | 19.10	4.48 | 5.85	2.03 | 3.33
20 °C (t_eq_)	11.68 | 13.08	3.08 | 4.05	0.90 | 2.72	15.40 | 19.10	4.48 | 5.85	2.03 | 3.33
30 °C (t)	3.32 | 3.82	1.08 | 1.48	0.65 | 1.07	9.02 | 10.27	1.82 | 2.40	1.35 | 1.98
30 °C (t_eq_)	9.19 | 10.58	2.93 | 4.01	2.07 | 3.39	25.93 | 29.52	4.99 | 6.59	4.14 | 6.08
